# Anthropometry, glucose homeostasis, and lipid profile in prepubertal children born early, full, or late term

**DOI:** 10.1038/srep06497

**Published:** 2014-09-29

**Authors:** José G. B. Derraik, Tim Savage, Harriet L. Miles, Fran Mouat, Paul L. Hofman, Wayne S. Cutfield

**Affiliations:** 1 Liggins Institute, University of Auckland, Auckland, New Zealand; 2 Gravida: National Centre for Growth and Development, Auckland, New Zealand; 3 Starship Children's Hospital, Auckland District Health Board, Auckland, New Zealand

## Abstract

To examine differences in growth and metabolism in prepubertal children born early term, full term, and late term. We retrospectively studied 294 prepubertal children aged 7.3 years (range 3.0–12.1 years). Children were separated into those born early term (37 0/7–38 6/7 weeks of gestation; n = 68), full term (39 0/7–40 6/7 weeks; n = 179), and late term (41 0/7–41 6/7 weeks; n = 47). Clinical assessments included anthropometry, DXA-derived body composition, fasting lipids, and glucose homeostasis. Statistical models accounted for important confounding factors, such as gender, age, birth weight SDS, birth order, and parental variables. When birth weight was adjusted for sex and gestational age (birth weight SDS), late terms were heavier than both early (p = 0.034) and full (p = 0.020) terms. Early term children were shorter than both full (p = 0.010) and late (p = 0.049) term children, but differences in height disappeared following correction for parents' heights. There were no differences in glucose homeostasis, BMI SDS, adiposity, or fat distribution between groups. Lipid profiles were also similar. When important confounding factors were accounted for, there were no meaningful differences in anthropometry, glucose homeostasis, and lipid profile among children born early term, full term, or late term.

The World Health Organization defines *term* gestation as those pregnancies ending between 37 completed weeks to less than 42 completed weeks of gestation^1^. Children born late preterm (34 0/7–36 6/7 weeks of gestation) have been found to have higher blood pressure^2^ and higher plasma insulin levels^3^ than those born at term. Considerable metabolic and auxologic differences were found in prepubertal children born post-term (≥42 0/7 weeks of gestation) compared to children born at term^4^. There is also increasing evidence that neonatal outcomes vary according to timing of delivery within the term window^5^,^6^. As a result, the American College of Obstetrics and Gynecology has recently recommended the revision of the classification of term gestations into *early term* (37 0/7–38 6/7 weeks of gestation), *full term* (39 0/7–40 6/7 weeks), and *late term* (41 0/7–41 6/7 weeks)^7^.

Studies have shown that babies born early term experience greater mortality and morbidity in the neonatal period and early infancy than those born full term^5^,^6^,^8^. A United States study on nearly 189,000 deliveries observed increased rates of neonatal intensive care unit admission and respiratory morbidity in early terms than in full terms^8^. A retrospective study of 46,329,018 singleton live births (also in the USA) showed that early term births were associated with higher neonatal and infant mortality than those born full term^6^.

There is particularly strong evidence of differences in neurocognitive outcomes. At one-year of age, mental and psychomotor developmental scores increased with increasing gestational age from 37 to 41 weeks of gestation among 1,500 infants in Chile^9^. A study on over 400,000 Scottish school-aged children born at term showed an increased incidence of special education need in those born early term^10^. Other large studies in the USA and Belarus have shown poorer academic performance and lower IQ in early term children^11^,^12^. Similar results were obtained for intellectual performance in over 300,000 Swedish men at conscription^13^.

However, there are no data on auxologic and metabolic outcomes in childhood in association with gestational age within the term window. As a result, we aimed to evaluate anthropometry, glucose metabolism, and lipid profile according to timing at delivery in prepubertal children born at term.

## Methods

### Ethics approval

Ethics approval for this study was provided by the Northern Y Regional Ethics Committee (Ministry of Health, New Zealand) and the University of Auckland Human Participants Ethics Committee. Written informed consent was obtained from parents or guardians, as well as verbal or written consent from each child as was appropriate to their age. This study was performed in accordance with all appropriate institutional and international guidelines and regulations for medical research, in line with the principles of the Declaration of Helsinki.

### Study cohort

We undertook a large project examining the effects of parental and prenatal factors in the offspring. From this larger project, we have examined the impact of conception with ovarian stimulation drugs on growth and metabolism in childhood^14^. Children conceived after ovarian stimulation were asked to invite 4–5 family friends and school friends who were naturally conceived to participate in the study as controls^14^, so that controls were recruited by study participants and of similar age, ethnicity, and socioeconomic status. In this study we retrospectively assessed the naturally conceived control cohort that was recruited between October 2010 and October 2012.

Only healthy, developmentally normal, prepubertal children aged 3–10 years, born 37–41 weeks of gestation were studied. Gestational ages were determined by ultrasound scans performed <20 weeks of gestation. All children were of New Zealand European ethnicity, naturally conceived, born of singleton pregnancies. All potential participants had pubertal development assessed by a paediatrician, and those with signs of puberty (Tanner stage 2 breast development in girls and testicular volume > 3 ml in boys or evidence of adrenarche) were excluded. Other exclusion criteria were being born small-for-gestational-age (<−2 standard deviation scores (SDS)), receiving medication that could affect insulin sensitivity or growth, or having a first degree relative with pre-diagnosed diabetes. Children were also excluded if born to mothers with gestational diabetes, chronic illnesses, or prolonged maternal drug use (including tobacco). All participants were of higher socioeconomic status according to their residential address and the “decile score” of the school they attended^15^.

### Clinical assessments

All clinical assessments were carried out by a single investigator at the Maurice & Agnes Paykel Clinical Research Unit (Liggins Institute, University of Auckland). Standing height was measured using a Harpenden stadiometer to the nearest mm. Children's weight and body composition were assessed using whole-body dual-energy X-ray absorptiometry (DXA Lunar Prodigy 2000; General Electric, Madison, WI, USA). Apart from total body fat percentage, other DXA-derived parameter of interest was the android fat to gynoid fat ratio (an indicator of abdominal adiposity).

Each child's birth weight, height, and BMI were transformed into SDS^16^,^17^. Maternal obstetric history was recorded to clarify parity and relevant medical history. Maternal and paternal height, weight, and body mass index (BMI) were measured. Parents' BMI were transformed into SDS, and the mean parental BMISDS (MPBMISDS) was calculated. Mid-parental height SDS (MPHSDS) was calculated^18^, and the child's height SDS was then individually corrected for their genetic potential (parents' heights), using the formula: “HtSDS – MPHSDS”.

Following an overnight fast, blood samples were drawn for assessment of total cholesterol, high-density lipoprotein cholesterol (HDL-C), low-density lipoprotein choleterol (LDL-C), and triglycerides. Children also had glucose and insulin levels measured, with insulin sensitivity evaluated using the homeostasis model assessment of insulin resistance (HOMA-IR)^19^.

### Assays

Plasma insulin was measured using an Abbott AxSYM system (Abbott Laboratories, Abbott Park, IL, USA) by microparticle enzyme immunoassay (Abbott Diagnostics, Wiesbaden, Germany) with an inter-assay coefficient of variation (CV) of <5%. Glucose, triglyceride, total cholesterol, HDL-C, and LDL-C concentrations were measured on a Hitachi 902 autoanalyser (Hitachi High Technologies Corporation, Tokyo, Japan) by enzymatic colorimetric assay (Roche, Mannheim, Germany) with an inter-assay CV of 1.2% for glucose, and <5% for the other parameters.

### Power calculation

The studied number of children in each term group provides 80% power at 5% level of significance (two-sided) to detect a difference of at least 25% in HOMA-IR between any two groups, assuming a standard deviation of 0.68 for children of this age^20^. The observed sample size also provides 80% power to detect a difference of 10% or more in HDL-C, assuming a standard deviation of 0.33 mmol/l^20^.

### Statistical analysis

Children were divided into three groups: early term, full term, and late term. Demographic data were compared using one-way ANOVA and Fisher's exact tests in Minitab v.16 (Pennsylvania State University, State College, PA, USA). Other comparisons between term groups were carried out using linear regression mixed models in SAS v.9.3 (SAS Institute, Cary, NC, USA), which included family identification number as a random factor to account for the clustering of siblings. All models accounted for important confounding factors, namely age, gender, birth weight SDS, birth order, and maternal age. Other factors were controlled for as required, depending on the outcome response of interest: for lipids and outcomes associated with glucose homeostasis – BMISDS; for anthropometric data – the appropriate parental factor (i.e. MPBMISDS or MPHSDS). Associations with study outcomes were also assessed with gestational age as a continuous variable. Demographic data are provided as means ± standard deviation; other data are means and 95% confidence intervals adjusted for confounders in the multivariate models.

## Results

A total of 343 children volunteered to participate in the original study; 294 were born at term and met the inclusion criteria. Participants were aged 7.3 ± 2.2 years (range 3.0–12.1 years).

Children in the three groups were of similar age and sex ratio (Table 1). However, there was a greater proportion of first-borns among late terms than in both early (p = 0.049) and full (p = 0.047) term groups (Table 1). As expected, there was a gradual increase in birth weight according to gestational age (Table 1). However, even when birth weight was adjusted for gender and gestational age (i.e. birth weight SDS), late terms were heavier than both early (p = 0.034) and full (p = 0.020) terms (Table 1).

Early term children were shorter than both full (p = 0.010) and late (p = 0.049) term children (Table 2). However, there were differences in mid-parental height SDS among groups (data not shown), so that when children's heights were individually corrected for parents' heights any significant differences disappeared (Table 2). There were no differences in BMI SDS, adiposity, or fat distribution among groups (Table 2).

There were also no differences in HOMA-IR, fasting glucose, or fasting insulin concentrations (Table 2). Lipid profiles were similar among the three groups, except that total cholesterol concentrations were higher in late term than in full term children (p = 0.035; Table 2).

Analyses examining gestational age as a continuous variable yielded nearly identical results. There was a positive association with height SDS (p = 0.014), which was no longer observed when children's heights were individually corrected for parents' heights.

## Discussion

This study showed that anthropometry, glucose homeostasis, and lipid profile were similar in prepubertal children born early, full, or late term. An observed difference in height disappeared after controlling for parental heights, and an isolated difference in cholesterol was likely a Type I error and was disregarded.

Even though we have not observed any metabolic or auxologic differences across the three term groups, other have found such differences on the flanking edges of the term range^2^,^3^,^4^. As previously discussed, a number of studies have shown that children born early term have poorer neurocognitive outcomes than children born full term^9^,^10^,^11^,^12^.

To our knowledge, this is the first study to examine both growth and metabolism according to the timing of delivery in children born at term. However, Wang et al. observed that in early childhood those born early term tended to have higher plasma insulin levels than children born full term^3^. Although we observed no differences prior to puberty, one cannot discard that there may be dissimilarities later in life. Yang et al. observed a subtle increase in systolic blood pressure in young men born early term^13^. However, there were no differences in height or BMI, and they did not examine fat distribution or metabolism (other than blood pressure)^13^. Further, Leger et al. found no association between gestational age and final adult height among adults born at term^21^.

Interestingly, we observed a greater proportion of first-borns among late terms than in both early and full term groups. This finding is in agreement with the observations of a previous study, which showed a small difference in gestational age (0.2 weeks) between first- and later-born children^22^.

The limitations of our study include our relatively small cohort and the uneven distribution of children among the three term groups. We studied a homogenous group of children (same ethnicity and higher socioeconomic status), which likely eliminated much of the phenotypic and metabolic variability associated with these factors. However, this homogeneous cohort also limits the application of our findings to the general population, particularly to those of lower socioeconomic status. Importantly, data from 81 singleton pregnancies after *in vitro* fertilization (where conceptions can be accurately timed) showed that ultrasound scans in the first 20 weeks underestimated gestational age by 2.8 days (standard error of the mean = 0.2), with fetal age determined to within 7 days in more than 95% of cases^23^. Thus, we acknowledge that a minority of participants could have had their grouping misclassified based on the ultrasound scans.

In summary, in contrast to the observed differences in neurodevelopmental outcomes according to gestational age among those born at term, there were no differences in anthropometry, glucose homeostasis, or lipid profile in prepubertal children. Larger studies in both childhood and adulthood are necessary to assess whether any such differences do exist among those born early, full, or late term.

## Author Contributions

J.G.B.D., T.S., W.S.C., P.L.H., H.L.M. and F.M. conceived and designed the study. T.S. performed the clinical assessments. T.S., H.L.M., F.M. and J.G.B.D. compiled the data, which were analysed by J.G.B.D.. J.G.B.D. wrote the manuscript with input from all other authors.

## Figures and Tables

**Table 1:** Demography of our study cohort according to gestational age at delivery. Data are means ± SD. *p < 0.05, ****p < 0.0001 vs late term; ††††p < 0.0001 vs full term

	Early term	Full term	Late term
**n**	68	179	47
**Sex ratio** (boys/girls)	40/28	88/91	28/19
**Birth order** (first-borns)	46%*	48%*	64%
**Age** (years)	7.1 ± 2.0	7.3 ± 2.2	7.7 ± 2.2
**Birth weight** (kg)	3.22 ± 0.46****††††	3.56 ± 0.42****	3.90 ± 0.45
**Birth weight SDS**	0.16 ± 0.96*	0.18 ± 0.92*	0.52 ± 0.94

**Table 2:** Study outcomes among prepubertal children separated according to gestational age at delivery. Data are means and 95% confidence intervals adjusted for all confounding factors in the multivariate models. *p < 0.05 vs late term; ^†^p < 0.05 vs full term

	Early term	Full term	Late term
**n**	68	179	47
**Auxology**			
Height SDS	0.61 (0.38–0.83)*†	0.93 (0.80–1.07)	0.94 (0.68–1.20)
Height SDS – MPHSDS	0.40 (0.21–0.58)	0.27 (0.15–0.39)	0.19 (−0.02–0.41)
BMI SDS	−0.11 (−0.36–0.14)	−0.03 (−0.19–0.12)	−0.21 (−0.51–0.09)
Total body fat (%)	16.7 (15.4–18.1)	16.8 (16.0–17.7)	16.8 (15.2–18.5)
Android fat to gynoid fat ratio	0.60 (0.56–0.64)	0.57 (0.55–0.60)	0.58 (0.54–0.63)
**Glucose homeostasis**			
HOMA-IR	1.05 (0.93–1.20)	1.06 (0.98–1.15)	1.05 (0.91–1.22)
Fasting glucose (mmol/l)	4.78 (4.68–4.87)	4.80 (4.74–4.86)	4.77 (4.66–4.88)
Fasting insulin (mU/l)	4.98 (4.43–5.61)	4.96 (4.62–5.34)	4.95 (4.32–5.66)
**Lipid profile**			
Total cholesterol (mmol/l)	4.32 (4.15–4.49)	4.29 (4.18–4.39)*	4.53 (4.32–4.74)
HDL-C (mmol/l)	1.33 (1.25–1.43)	1.38 (1.33–1.43)	1.44 (1.34–1.53)
LDL-C (mmol/l)	0.89 (0.82–0.95)	0.88 (0.85–0.92)	0.95 (0.88–1.02)
Total cholesterol to HDL-C ratio	3.78 (3.16–3.60)	3.32 (3.18–3.46)	3.30 (3.04–3.56)
Triglycerides (mmol/l)	0.72 (0.66–0.78)	0.72 (0.68–0.76)	0.73 (0.66–0.81)
